# Remodeling of the circRNA Landscape in Myocardial Infarction Integrates Nuclear Regulation, DNA Damage Response, and Cardiomyocyte Structural Pathways

**DOI:** 10.3390/biom16040578

**Published:** 2026-04-14

**Authors:** Rudaynah Alali, Naif Khalid Alqannas, Alawi H. Habara, Mohammed Almansori, Ali Alsaeed, Chittibabu Vatte, Cyril Cyrus, Safi G. Alqatari, Hassan Albisher, Mustafa H. Al-ajwad, Faisal S. Alshahrani, Moyad M. Almuslim, Morten T. Venø, Brendan J. Keating, Amein K. Al-Ali

**Affiliations:** 1 Department of Internal Medicine, Cardiology Section, King Fahd Hospital of the University, Alkhobar 34445, Saudi Arabia; raali@iau.edu.sa (R.A.); mmansori@iau.edu.sa (M.A.); 208019919@iau.edu.sa (M.H.A.-a.); fsnshahrani@iau.edu.sa (F.S.A.); 2 Imam Abdulrahman bin Faisal University, Dammam 31441, Saudi Arabia; sagqatari@iau.edu.sa (S.G.A.); hmalbisher@iau.edu.sa (H.A.); 3 Department of Adult Cardiology, Saud Al Babtain Cardiac Center, Dammam 32245, Saudi Arabia; naifkhalid@gmail.com; 4 Department of Biochemistry, College of Medicine, Imam Abdulrahman bin Faisal University, Dammam 31441, Saudi Arabia; ahhabara@iau.edu.sa (A.H.H.); cbvatte@iau.edu.sa (C.V.); ccyrus@iau.edu.sa (C.C.); 5 Dammam Medical Complex, Dammam 32245, Saudi Arabia; alih6234@gmail.com; 6 Department of Internal Medicine, Rheumatology Unit, King Fahd Hospital of the University, Alkhobar 34445, Saudi Arabia; 7 Department of Surgery, King Fahd Hospital of the University, Alkhobar 34445, Saudi Arabia; 8 College of Pharmacy, Imam Abdulrahman bin Faisal University, Dammam 31441, Saudi Arabia; mmalmuslim@iau.edu.sa; 9 Omics ApS, Åbogade 15, 8200 Aarhus, Denmark; morten.veno@omiics.com; 10 Department of Surgery, NYU Grossman School of Medicine, 530 First Avenue, Suite 9V, New York, NY 10016, USA; brendan.keating@nyulangone.org

**Keywords:** RNASeq, circRNA, MI, biomarker, inflammation

## Abstract

Plasma circular RNAs (circRNAs) are stable RNA molecules found in blood, which makes them potential noninvasive biomarkers for acute myocardial infarction (MI). The aim of this study was to describe the plasma circRNA profile in patients with acute MI and to identify circRNA markers that may help detect heart injury and reflect the biological processes involved. We compared plasma samples from patients with acute MI and healthy controls using total RNA sequencing with unique molecular identifiers (UMIs). After sequencing, reads were processed through quality control, alignment, duplicate removal, and circRNA detection. Differential expression was analyzed after adjusting for age, sex, smoking, and technical factors. Several circRNAs were significantly different between MI cases and controls and were able to separate the two groups in principal component and receiver operating characteristic analyses. Among the most increased circRNAs were hsa-PASK_0004, hsa-STXBP3_0002, hsa-RCAN3_0002, and hsa-RANBP9_0044, while hsa-HIF1A_0002, hsa-SUZ12_0049, hsa-PNRC1_0001, and hsa-RAB2A_0002 were decreased. Several candidates showed AUC values above 0.7. Pathway analysis linked the host genes of these circRNAs to inflammation, platelet activation, coagulation, and cardiomyocyte stress responses. Overall, these findings suggest that circulating circRNAs may serve as useful blood-based markers of MI and provide insight into the molecular changes that accompany acute MI.

## 1. Introduction

Myocardial infarction (MI) is a leading cause of morbidity and mortality worldwide, representing the most severe acute consequence of atherosclerosis, a chronic inflammatory arterial disease in which lipid-rich plaques accumulate, destabilize, and rupture or erode to trigger occlusive coronary thrombosis and myocardial ischemia [1]. Despite effective reperfusion therapies, residual risk remains substantial, underscoring the need to develop novel diagnostic biomarkers that can detect myocardial injury earlier and with greater specificity than current assays. In recent years, non-coding RNAs have been recognized as key regulators of cardiovascular pathophysiology. Among these, circular RNAs (circRNAs) have emerged as a class of covalently closed, single-stranded RNA molecules generated by back-splicing, characterized by exceptional stability in plasma, evolutionary conservation, cell-type specificity, and context-dependent functions [2,3,4,5]. Circulating circRNAs are detectable in plasma and reflect changes in people with heart disease compared with healthy individuals and this makes them potential, non-invasive biomarkers for detection and progression of MI [6].

circRNAs display diverse molecular activities, acting as microRNA sponges or decoys, scaffolding RNA-binding proteins, modulating transcription and splicing, interacting with chromatin, and, in some cases, encoding micropeptides via Internal Ribosome Entry Site—or m6A-driven translation [7,8]. Consequently, circRNAs are adaptable regulators of gene expression relevant to cardiomyocyte survival, endothelial function, vascular smooth muscle action, inflammatory signaling, and fibroblast activation [9]. Critically, their circular topology confers resistance to exonucleases, enabling persistence in biofluids and extracellular vesicles. This exceptional plasma stability, combined with disease- and tissue-specificity, positions circRNAs as particularly attractive candidates for blood-based diagnostic biomarkers that may offer earlier and more specific detection of myocardial injury than conventional protein markers.

Stressors including hypoxia, hemodynamic overload and oxidative stress alter the expression of circRNAs [10]. Studies in human tissues, animal models, and cell systems reveal MI-associated circRNA signatures that correlate with infarct size, left ventricular function, reperfusion injury, and clinical outcomes [11,12]. Specific circRNAs have been implicated in pathways relevant to MI pathogenesis, including mitochondrial homeostasis, necroptosis, apoptosis, angiogenesis, autophagy, immune cell recruitment, and extracellular matrix deposition [10,13]. Several candidates demonstrate diagnostic and prognostic potential in plasma or serum, complementing established markers such as troponins and natriuretic peptides [11,14]. Functional studies in animals and lab models using methods that either increase or block circRNAs suggest that these molecules can influence how the heart heals after an infarct and may contribute to harmful long-term remodeling [15]. These results push researchers to look into treatments that target circRNAs—either by blocking the harmful ones or bringing back the helpful ones. This could be done using tools like antisense drugs, siRNA, CRISPR-style gene editing, or by packing the therapy into engineered exosomes to deliver it to cells [16,17]. Translational utilization of circRNAs faces several challenges [16]. These include technical variability in detection and quantification, the need for robust normalization and isoform-level resolution, and the contextual nature of circRNA–miRNA–protein interactions, all of which complicate discovery and validation [18,19]. Important and current investigation areas include distinguishing circRNAs from byproducts of splicing, guaranteeing cardiac and cell-type specificity, optimizing delivery, and minimizing off-target effects [18,20]. In addition, manufacturing and regulatory pathways for RNA therapy need to have strong durability, safety and biodistribution profiling [20,21].

However, this area of research is still very new, particularly when it comes to cardiovascular disease. Thus this study interrogates circRNA biology in a clinical setting by comparing circulating and myocardial circRNA profiles between patients with acute MI and a demographically matched control group. We used RNA-seq and orthogonal validation to identify circRNAs that were differentially expressed [22]. Using established knowledge of circRNA biogenesis, function, and stability, we carefully evaluated potential case–control biases arising from isoform-level detection, sample processing, and normalization. Building on prior reports linking circRNAs to ischemic injury, cardiomyocyte stress responses, and adverse remodeling, we sought to test whether distinct circRNA signatures discriminate MI cases from healthy controls and reflect infarct biology. Our two primary objectives in this study were to firstly define MI-associated circRNA expression changes and secondly to evaluate the diagnostic and prognostic potential of circRNA using both unsupervised clustering and ROC curve analyses (AUCs) benchmarks to clinical measures.

Together, this work highlights circRNAs linked to nuclear regulation, DNA damage response, and cardiomyocyte structural pathways in MI and provides a focused set of candidates for follow-up validation and functional studies.

## 2. Materials and Methods

### 2.1. Study Design and Participants

This was a prospective observational case–control study enrolling adults with acute MI and demographically matched controls from King Fahd Hospital of the University (Al-Khobar) and Dammam Medical Complex (Dammam), both located in the Eastern Province of Saudi Arabia. Eligible cases were patients presenting with MI as defined by ACC/AHA recommendations and the Fourth Universal Definition of Myocardial Infarction, based on a rise and/or fall in cardiac troponin with at least one value above the 99th percentile upper reference limit, together with evidence of acute myocardial ischemia, such as ischemic symptoms, new ischemic ECG changes, pathological Q waves, or imaging evidence of new loss of viable myocardium [1]. Given that MI in this setting predominantly reflects underlying atherosclerotic coronary artery disease, our cohort captures a clinically relevant manifestation of atherothrombosis. Controls were individuals without a history of MI who were frequency-matched to cases by sex, and major cardiovascular risk factors, and were recruited from the same centers and catchment area to minimize selection bias. All participants provided written informed consent before enrollment.

Participants were adults aged 21–88 years with a clinical diagnosis of MI at the time of recruitment. Because longitudinal medical records were incomplete for many older potential controls, we could not reliably exclude prior myocardial infarction or clinically significant coronary disease in that group. Therefore, we preferentially recruited younger controls to minimize control-group misclassification, while recognizing that this introduces an age imbalance that may contribute to observed differences.

Phenotypic data for all participants was reviewed by a consultant cardiologist to ensure consistency across sites and confirm eligibility. Cases with uncertain phenotypic classification were referred to a consultants’ committee for adjudication; additional clinical information was requested, when necessary, prior to a final inclusion decision.

Inclusion criteria comprised any of the following: presentation with MI, documented coronary artery disease (CAD), or prior coronary revascularization (percutaneous coronary intervention or coronary artery bypass grafting). Exclusion criteria included chronic obstructive pulmonary disease, right-sided heart failure, chronic kidney disease (CKD) requiring ongoing management, active or prior malignancy, nonspecific or atypical chest pain without objective evidence of ischemia, and first-degree relatives of individuals already enrolled.

Blood samples were obtained in EDTA tubes, within two hours of the index MI event. Plasma was separated within one hour of phlebotomy and stored at −80 °C until RNA sequencing. The study was approved by the Institutional Review Board of Imam Abdulrahman bin Faisal University (IRB# 2019-01-104) and the Ministry of Health, Dammam (IRB# 2023-02, 29 March 2023). All procedures adhered to the principles of the Declaration of Helsinki. Written informed consent was obtained from all participants prior to enrollment.

### 2.2. Plasma RNA Isolation and Library Preparation

Plasma total RNA was isolated from 500 µL plasma per sample using the miRNeasy serum/plasma advanced kit (Qiagen, Germantown, MD, USA) and treated with TURBO DNA-free™ Kit (Thermo Fisher, Waltham, MA, USA). Ribosomal RNA was depleted and stranded total RNA libraries prepared using SMARTer^®^ Stranded Total RNA-Seq Kit v3 Pico Input Mammalian (Takara, Kusatsu, Shiga, Japan), which includes unique molecular indices (UMIs) for PCR duplicate removal. Library QC was performed on an Agilent Bioanalyzer 2100 (California, USA), equimolarly pooled and sequenced on an Illumina NextSeq (California, USA) (paired end 150 bp).

#### Preprocessing, Mapping, UMI Deduplication and Quantification

Adapters and low-quality bases (Phred score < 20) were removed using Trim Galore. Read QC was assessed with FastQC and multiQC; samples with low complexity or poor QC metrics were excluded. Reads were aligned to hg19/GRCh37 using STAR. UMIs were used for PCR duplicate removal via UMI-tools. Gene counts were produced with featureCounts against Gencode v37 annotations. For circRNA detection, deduplicated fastq files were processed with PRINSEQ lite and circular junctions were identified primarily with CIRI2 and secondarily with find_circ. Identified circRNAs were cross-referenced with circBase, CIRCpedia and circAtlas.

To support the reliability of circRNA detection and to prioritize candidates for downstream analyses, we performed circRNA-specific in silico quality and coherence analyses. First, we compared back-splice junction read support for each circRNA to linear spliced read support in the corresponding genomic region (linear-versus-circular splice support plot). Second, we assessed concordance between circRNA expression and expression of the corresponding host-gene mRNA using Pearson correlation across samples. Third, we compared group-wise expression changes for circRNAs to the changes observed for mRNAs from the same host genes (delta linear versus delta circular expression). Together, these analyses help distinguish circRNAs with strong back-splice support and circRNA-specific regulation from changes that simply mirror host-gene mRNA expression; experimental validation in independent cohorts and with junction-specific assays remains a priority for future work.

### 2.3. Differential Expression and Statistical Modeling

Differential expression was performed with DESeq2 (R) using the model:

design = ~Sex + Smoking + Batch + Age + Percent_mRNA_mapping + Group

This model adjusts for demographic/technical confounders; percent reads mapping to gene exons served as a sample quality covariate. We combined linear gene counts and circRNA backsplice counts in DESeq2 where appropriate (host gene interpretation was based on circRNA host gene mapping). Multiple testing correction used the Benjamini–Hochberg FDR; FDR < 0.05 was considered significant unless otherwise noted.

### 2.4. Biomarker Performance

Receiver operating characteristic (ROC) curves and AUCs were generated using the pROC package. Candidate biomarkers were selected based on effect size (log2 fold change), adjusted *p*-value, expression robustness across samples, presence in external circRNA databases (for circRNAs), and AUC performance.

### 2.5. Data Visualization and Software

All statistical analyses and data visualizations were carried out in R using scripted workflows to ensure reproducibility.

## 3. Results

The clinical and demographic characteristics of the study population are summarized in Supplementary Table S1.

**Figure 1:**
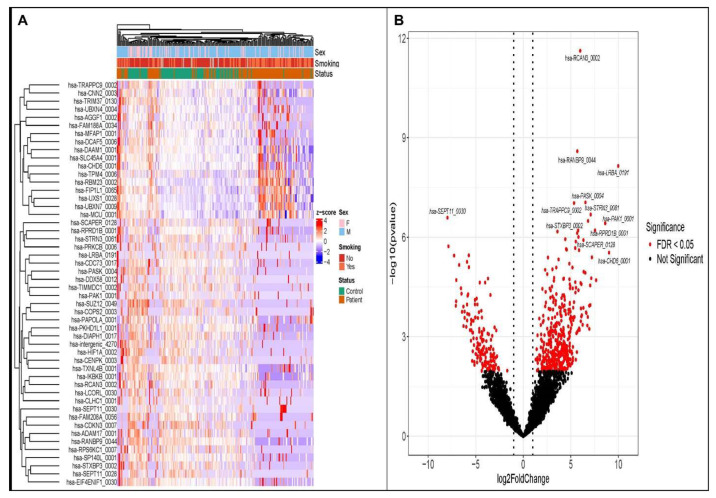
Plasma circRNA differential expression and sample-level clustering after myocardial infarction (MI). (**A**): Heatmap of expression patterns for the top differentially expressed circRNAs across all samples. Rows represent circRNAs and columns represent individual samples. Expression values are shown as row-wise Z-scores. Unsupervised hierarchical clustering was applied to both rows and columns. Annotation bars above the heatmap indicate sex, smoking status, and clinical group status (control or patient). (**B**): Volcano plot showing differential circRNA expression between MI cases and healthy controls. The x-axis represents log2 fold change and the y-axis represents −log10 *p* value. Red points indicate significantly differentially expressed circRNAs at FDR < 0.05, while black points represent non-significant circRNAs. Selected circRNAs with prominent changes are labeled.

The study cohort consists of 140 clinically confirmed MI cases and 109 healthy control subjects with the mean age of 55.1 and 20.17 years respectively. Among the patient cohort, 62.1% were presented with hypertension, 60.7% were smokers and had type 2 diabetes mellites (Supplementary Table S1). Among the control subjects only 8.25% were smokers and none had a history of diabetes and/or hypertension. Differential expression analysis of circRNAs was performed on RNA-seq data of MI patients and healthy control subjects using DESeq2. Global analysis of the circRNA landscape identified 607 circRNAs that were significantly differentially expressed in MI, including 441 upregulated and 166 downregulated. The volcano plot depicting all detected circRNAs demonstrated a broad distribution of log2 fold changes, with a clear subset of circRNAs showing large-magnitude changes and strong statistical significance (Figure 1A). Volcano plots for the upregulated and downregulated circRNAs highlighted that, while a prominent cluster of circRNAs was markedly increased in MI, a distinct set was similarly strongly suppressed, suggesting coordinated activation and repression of circRNA, producing loci in response to myocardial injury. Significantly dysregulated circRNAs (false discovery rate-adjusted *p* < 0.05) were enriched among both up- and down-regulated species, indicating a bidirectional remodeling of the circular transcriptome in MI.

To delineate the most prominent candidates, we ranked circRNAs by statistical significance and effect size. The top 50 differentially expressed circRNAs, including both up- and down-regulated transcripts, are summarized in Supplementary Tables S2 and S3. The number of circRNAs and their level of expression based on chromosome location are depicted in Supplementary Figure S1. These circRNAs mapped predominantly to protein-coding host genes and encompassed exonic circRNAs with a range of genomic loci, including genes previously implicated in CVD/CAD-related biology and cellular stress responses.

Many of the most strongly upregulated circRNAs including has-PASK_0004 (*p* < 0.001; AUC up to 0.745), hsa-STXBP3_0002 (*p* < 0.001; AUC up to 0.751), hsa-RCAN3_0002 (*p* < 0.001; AUC up to 0.715), and hsa-RANBP9_0044 (*p* < 0.001; AUC up to 0.812) stood out because they showed very large expression shifts with log2 fold changes greater than 5 and also promising diagnostic performance as reflected by AUC values from ROC analyses. The presence of multiple supporting back-splice junction reads further supports that these signals reflect robust induction in MI. In contrast, several circRNAs including hsa-HIF1A_0002 (*p* < 0.001; AUC up to 0.812), hsa-SUZ12_0049 (*p* < 0.001; AUC up to 0.803), hsa-PNRC1_0001 (*p* < 0.001; AUC up to 0.857), and hsa-RAB2A_0002 (*p* < 0.001; AUC up to 0.831) were among the most downregulated based on large negative log2 fold changes together with strong ROC AUC performance, consistent with a disease-associated reduction in specific circular isoforms. The area under the ROC curve (AUC) was calculated as a measure of the discriminatory power of circRNAs, showing their ability to differentiate between MI patients and controls. The AUC values are listed for the 50 differentially up- and down-expressed circRNAs in Supplementary Tables S2 and S3. Collectively, these data nominate a set of candidate circRNAs, hsa-PASK_0004, hsa-STXBP3_0002, hsa-RCAN3_0002 and hsa-RANBP9_0044 which are upregulated and hsa-HIF1A_0002, hsa-SUZ12_0049, hsa-PNRC1_0001, and hsa-RAB2A_0002 which are down regulated that are tightly linked to the MI phenotype and warrant further functional investigation.

**Figure 2:**
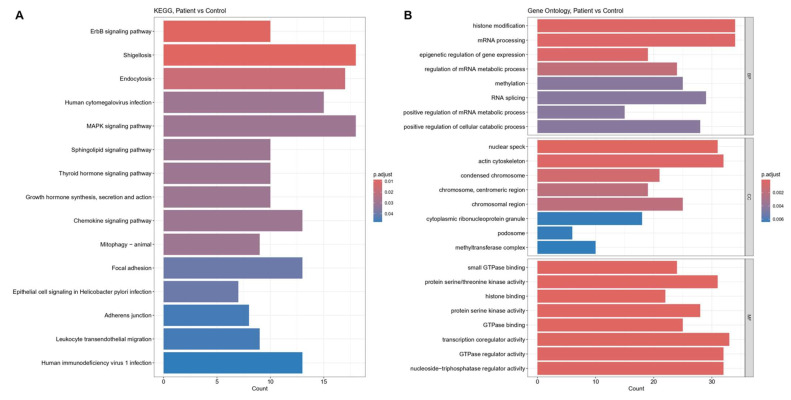
Functional enrichment of circRNA host and target genes (KEGG and GO analyses). (**A**) Main panel: Bar plot or dot plot (as shown in the figure) summarizing the top KEGG pathways enriched among target/host genes of differentially expressed circRNAs (nominal *p* values shown). Pathways are grouped into higher-level classifications (Core cardiovascular & ischemic injury pathways; Cellular stress, metabolism, and vascular signaling; Systemic hormonal modulators) and ordered by significance. Gene counts mapped to each pathway are indicated by point size (or bar length), and color indicates nominal *p* value (gradient from low to high). (**B**) Inset/lower panel: Representative Gene Ontology (GO) terms for Biological Process (BP) and Molecular Function (MF) enriched among circRNA host genes, with terms such as histone binding and histone methyltransferase activity highlighted. Key driver genes (for example, CHD2, PHF14, PHIP, SMARCC1, DEK, SUZ12, KMT2C) are annotated where they contribute to multiple terms.

The functional context of these MI-associated circRNAs was investigated by conducting Gene Ontology (GO) and Kyoto Encyclopedia of Genes and Genomes (KEGG) pathway (Figure 2). Significant cardiovascular disease-related KEGG pathways identified based on the target genes for the differentially expressed circRNAs are listed in Table 1.

**Figure 3:**
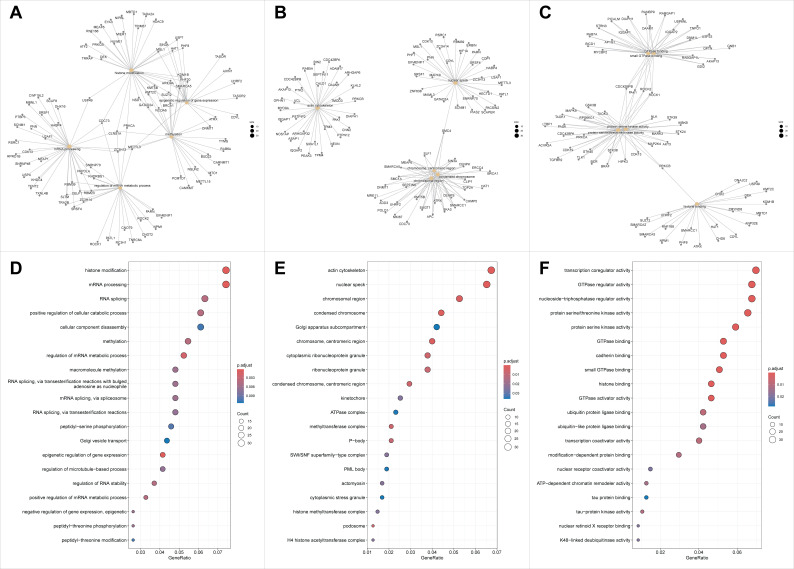
Functional enrichment networks and GO dot plots for host genes associated with dysregulated circRNAs. (**A**): Category network showing relationships between enriched GO terms and their member genes. Central nodes represent enriched functional categories and connected nodes represent genes assigned to each term. (**B**): Category network showing a second cluster of enriched GO terms and associated genes, with emphasis on nuclear, chromosomal, and cytoskeletal processes. (**C**): Category network showing a third cluster of enriched GO terms and associated genes, including molecular functions related to small GTPase binding, kinase activity, and histone binding. (**D**): Dot plot of enriched GO terms showing gene ratio on the x-axis, number of mapped genes by dot size, and adjusted *p* value by color. (**E**): Dot plot of enriched GO cellular component terms. (**F**): Dot plot of enriched GO molecular function terms.

The GO-based enrichment analyses of biological processes (BP), cellular components (CC), and molecular functions (MF) were explored using the host genes of significantly regulated circRNAs. The initial analysis showed that the host genes of upregulated circRNAs were significantly enriched for chromatin- and DNA-associated functions. The category network plot highlighted histone binding and histone methyltransferase activity as the dominant and most interconnected terms, with key chromatin regulators such as CHD2, PHF14, PHIP, SMARCC1, DEK, SUZ12, and KMT2C occupying central positions in the network. These results suggest that circRNAs significantly regulated in MI preferentially arise from genes governing epigenetic regulation, genome maintenance, and pro-survival signaling pathways (Figure 3).

Finally, unsupervised hierarchical clustering based on markedly dysregulated circRNAs showed an overall tendency for MI patients to group separately from control subjects (Figure 1B). While some overlap was observed, the circRNA expression heatmap indicated that many MI samples clustered together and differed from controls, suggesting that MI is associated with a reproducible circRNA expression pattern. Notably, several of the top differentially expressed circRNAs identified by DESeq2 (Supplementary Tables S2 and S3) contributed prominently to the clustering structure, supporting their potential as candidate biomarkers. Importantly, ROC analyses identified 62 circRNAs with AUC > 0.7 (Supplementary Tables S2 and S3), supporting their ability to discriminate MI patients from controls.

## 4. Discussion

In this study, we profiled the plasma circRNA landscape in acute MI, the most severe clinical consequence of atherosclerotic plaque rupture, using RNA sequencing of plasma samples from MI patients and healthy controls. We used differential expression analysis with functional enrichment to obtain a comprehensive picture of how circRNAs are remodeled in the acute atherothrombotic setting. These findings support circRNAs as both potential biomarkers of myocardial injury and as molecular clues to the biological processes active at the time of infarction.

In line with earlier studies showing that circRNAs are common, stable, tissue-specific, and able to regulate gene activity, and with reports that ischemic injury reshapes the non-coding RNA landscape, we identified a clear MI-associated circRNA signature, with some circRNAs increasing and others decreasing [13,14,23,24]. Overall, these results build on and extend previous cardiovascular research by expanding the list of MI-associated circRNAs and systematically linking them to specific biological pathways and regulatory networks [25,26].

An important finding was the disruption of circRNAs involved in hypoxia-related pathways, including HIF1A. The circRNA, hsa-HIF1A_0002 was significantly altered in MI patients (*p* < 0.001; AUC up to 0.812), suggesting that circulating circRNAs reflect the low-oxygen response after infarction. These findings show that circRNAs from HIF1A and other hypoxia-related genes are altered after MI, in line with ischemia–reperfusion-related oxidative stress and metabolic dysfunction in cardiomyocytes [27,28,29]. Several downregulated circRNAs were also linked to SUZ12 and related chromatin regulators. Because SUZ12 is a key component of the Polycomb Repressive Complex 2, these findings suggest that epigenetic reprogramming after MI is reflected in the circulating circRNA pool [30]. Host-gene enrichment analysis further showed that the dysregulated circRNAs were linked to pathways involved in myocardial stress and remodeling, including inflammatory signaling, extracellular matrix organization, and cell-death processes. These pathways are closely related to the clinical course after MI, from infarct expansion and scarring to adverse remodeling and progression to heart failure, suggesting that circRNA changes in these pathways may also have clinical value [31,32]. Several dysregulated circRNAs also contained predicted miRNA-binding sites, and some of the related miRNAs have previously been linked to cardiac hypertrophy, fibrosis, inflammation, and cardiomyocyte death. Although these predictions still need experimental validation, they help identify circRNAs for future functional studies. Beyond these biological insights, the overall circRNA expression pattern could partly distinguish MI patients from healthy controls in unsupervised clustering analysis. ROC curve analysis further showed that several candidates achieved AUC > 0.7, supporting their diagnostic value in acute MI. Together, these findings suggest that circRNA profiles could complement existing clinical tools in ischemic heart disease by adding molecular information beyond current protein biomarkers and imaging, particularly in relation to atherosclerotic plaque biology. While earlier studies have highlighted individual circRNAs in plasma or cardiac tissue in coronary artery disease, our results support the value of examining broader circRNA expression patterns in MI. Overall, these findings suggest that circRNA profiles may have both biological and clinical relevance in MI. Future studies should confirm these circRNA changes in independent MI cohorts and examine whether they are associated with HIF1A expression in heart tissue, circulating hypoxia markers, or epigenetic regulatory pathways [33].

Another important finding was that MI-related circRNA changes were enriched in host genes involved in chromatin regulation, DNA replication, DNA damage response pathways, and broader genome maintenance programs. Although our data do not directly show increased DNA-repair activity, they do show consistent downregulation of circRNAs arising from key replication and repair genes. These included hsa-POLB_0016, hsa-TOP3A_0006, hsa-BRCA1_0011, and hsa-MRE11A_0003, derived from POLB, TOP3A, BRCA1, and MRE11, respectively, in MI patients. Rather than pointing to a simple increase in repair processes, this pattern suggests altered circular RNA output from genes that help preserve genomic integrity. This is in line with oxidative stress, mitochondrial dysfunction, and genomic instability in cardiomyocytes exposed to ischemia–reperfusion injury [34,35]. Overall, these findings suggest disruption of replication and DNA damage response networks after MI and identify specific host genes, including MRE11, TOP3A, BRCA1, and POLB, for further mechanistic study. Future studies should examine whether these circRNA changes reflect altered host gene expression, changes in alternative splicing or backsplicing, or cell-type-specific loss of repair capacity after injury [35,36].

A key strength of our study is that the pathways linked to the top 50 increased and decreased circRNAs are biologically relevant to myocardial infarction. Rather than showing random changes, these genes were involved in pathways closely related to heart injury, including gene regulation, DNA damage response, low-oxygen stress, inflammation, platelet activation, blood clotting, and changes in heart muscle structure. Together, these findings suggest that circulating circRNAs may not only serve as useful biomarkers, but may also reflect the main biological changes that occur during early heart damage and remodeling after myocardial infarction.

This study also has several limitations that should be considered. The MI and control groups differed substantially in age, which could influence circulating RNA profiles even after statistical adjustment. In addition, because this was an observational study, the plasma circRNA changes may reflect the effects of tissue injury or altered RNA clearance rather than direct drivers of disease. Another limitation is that plasma-based measurements do not show the exact cellular source of these circRNAs. They may reflect downstream consequences of myocardial injury, systemic inflammatory or thrombotic responses, changes in circulating blood cells, or altered RNA release and clearance rather than direct drivers of disease. Therefore, the biological interpretation of circulating circRNA signals should be made with caution, as plasma measurements alone cannot distinguish tissue-specific release from broader systemic responses. Future studies combining plasma and tissue profiling, or using cell-type-specific methods, will help clarify the origin of these circRNAs and support follow-up functional studies.

Although age was considered in the analysis, closer age matching between cases and controls would further reduce potential confounding and make the findings easier to interpret. In future studies, this could be addressed by recruiting age-matched controls, using frequency matching during enrollment, or selecting controls from a larger pool to better reflect the demographic profile of the MI group.

In addition, although several circRNAs showed promising diagnostic performance, the observed discrimination was moderate and was evaluated only in this discovery cohort. The absence of validation in larger, independent and clinically relevant cohorts, including patients with stable coronary artery disease, limits the generalizability of these findings. Longitudinal sampling would also be important to define the temporal dynamics of circRNA changes and to compare them with established biomarkers such as high-sensitivity troponin. Independent confirmation of key circRNA candidates using orthogonal methods such as RT-qPCR would further strengthen the robustness and reproducibility of the findings.

In addition to helping with diagnosis, circRNAs may also be useful for predicting outcomes after myocardial infarction. Future studies may show whether they are linked to infarct size, cardiac remodeling, worsening heart failure, or recurrent ischemic events. Some circRNAs may also have potential as treatment targets if they are directly involved in processes such as myocardial injury, inflammation, fibrosis, endothelial dysfunction, or thrombosis. The circRNA-miRNA interaction is especially important, because circRNAs can influence gene regulation by interacting with miRNAs. Future studies combining circRNA, miRNA, and mRNA profiling with functional experiments will help determine whether these molecules are only biomarkers or also potential therapeutic targets.

Despite these limitations, our findings also point to clear next steps for clinical translation. The most promising circRNAs should be tested in independent, well-characterized cohorts, including age- and comorbidity-matched patients with stable coronary artery disease. Serial sampling from symptom onset through recovery would also help define how circRNA changes compare over time with established markers such as high-sensitivity troponin. Because circRNAs are highly stable in plasma, they may be good candidates for clinical assay development. If validated, a circRNA-based panel could support earlier and more specific detection of MI, especially in patients with unclear troponin results or non-diagnostic ECGs, while complementing existing clinical tools.

## 5. Conclusions

This study provides a broad overview of plasma circRNA changes in acute MI. Several of the identified circRNAs were linked to biological pathways relevant to MI, including gene regulation, DNA damage response, inflammation, platelet and coagulation pathways, and cardiomyocyte remodeling. We also identified candidate circRNAs that were able to distinguish MI patients from controls in this discovery cohort. However, these findings should be considered preliminary, as they require validation in larger independent and clinically relevant cohorts, as well as confirmation using orthogonal methods. In addition, mechanistic studies will be necessary to clarify the biological origin and functional relevance of these circRNAs in the context of myocardial infarction. Together, our results support further investigation of circulating circRNAs as potential biomarkers of MI, but they do not yet establish clinical utility.

## Figures and Tables

**Table 1:** Significant cardiovascular disease-related KEGG pathways identified from target genes of differentially expressed circular RNAs (circRNAs). Pathway enrichment analysis was performed using the Kyoto Encyclopedia of Genes and Genomes (KEGG) database on the set of target genes corresponding to differentially expressed circRNAs. The table reports significantly enriched cardiovascular disease-related pathways, including pathway annotation, gene count, and significance metrics. Statistical significance was defined according to the reported adjusted threshold (typically FDR < 0.05). Differentially expressed circRNAs were defined using the study-specific expression and significance criteria described in Methods.

S. No	Classification	Pathways	Pathway ID	*p* Value	Genes
1	Core cardiovascular & ischemic injury pathways	MAPK signaling pathway	hsa04010	0.0295	PAK1, PRKCB, IKBKB, MAPK9, AKT3, TAOK3, PTPN7, PRKCA, RAPGEF2, TAOK1, RAP1B, TGFBR2, MAX, ATF2, ANGPT1, MAP2K4, BRAF, NLK
2		Chemokine signaling pathway	hsa04062	0.0297	PAK1, PRKCB, IKBKB, GNAQ, GSK3B, GNB1, AKT3, PTK2, ROCK2, ROCK1, RAP1B, PLCG2, BRAF
3		Leukocyte transendothelial migration	hsa04670	0.046	PRKCB, CD99L2, PRKCA, PTK2, ROCK2, ROCK1, RAP1B, PLCG2, VCL
4		Focal adhesion	hsa04510	0.0405	PAK1, DIAPH1, PRKCB, MAPK9, GSK3B, AKT3, PRKCA, PTK2, ROCK2, ROCK1, RAP1B, VCL, BRAF
5		Adherens junction	hsa04520	0.046	PTPRJ, ROCK2, ROCK1, RAP1B, TGFBR2, VCL, IQGAP1, NLK
6	Cellular stress, metabolism, and vascular signaling	Mitophagy	hsa04137	0.0298	HIF1A, CCZ1B, MAPK9, HUWE1, RAB5A, OPTN, MFN2, NBR1, RAB7A
7		Endocytosis	hsa04144	0.0164	MDM2, DNM3, ARFGEF2, SNX2, SH3GLB1, NEDD4L, ASAP2, RAB5A, TGFBR2, IST1, EPS15, ASAP1, RAB7A, RAB4A, CHMP5, ARFGAP3, VPS35
8		Sphingolipid signaling pathway	hsa04071	0.0297	PRKCB, GNAQ, MAPK9, SGMS1, AKT3, PRKCA, ROCK2, ROCK1, PPP2R2D, ABCC1
9		ErbB signaling pathway	hsa04012	0.0084	PAK1, PRKCB, MAPK9, GSK3B, AKT3, PRKCA, PTK2, PLCG2, MAP2K4, BRAF
10	Systemic hormonal modulators	Thyroid hormone signaling pathway	hsa04919	0.0297	HIF1A, PRKCB, MDM2, GSK3B, AKT3, PRKCA, PLCG2, SIN3A, NCOA2, MED12L
11		Growth hormone synthesis, secretion, and action	hsa04935	0.0297	PRKCB, GNAQ, MAPK9, GSK3B, AKT3, PRKCA, PTK2, PLCG2, ATF2, MAP2K4

## Data Availability

The datasets generated during the current study are available in the European Nucleotide Archive (ENA) repository https://www.ebi.ac.uk/ena/browser/view/PRJEB104626 (accessed on 12 April 2026), with accession number PRJEB104626. All requests for data can be sent to the corresponding author (AKA) and verified academic investigators will be granted full access.

## Supplementary Materials

The following supporting information can be downloaded at: https://www.mdpi.com/article/10.3390/biom16040578/s1, Supplementary Figure S1. Chromosomal distribution of differentially expressed circular RNAs (circRNAs). The figure shows the number of significantly upregulated and downregulated circRNAs mapped to host genes across individual chromosomes. Bars represent counts of circRNAs (not genes), categorized by direction of differential expression and chromosomal location. Differential expression significance thresholds were applied as described in the Methods. Supplementary Table S1. Case–control demographic and clinical characteristics. Supplementary Table S2. Top 50 upregulated circular RNA and their associated genes. Supplementary Table S3: Top 50 downregulated circular RNA and their associated genes.

## Abbreviations

The following abbreviations are used in this manuscript:

MI	Myocardial infarction
CVD	Cardiovascular disease
CAD	Coronary artery disease
circRNA	Circular RNA
RNASeq	RNA sequencing
miRNA	Micro RNA
UMIs	unique molecular identifiers
CRISPR	Clustered Regularly Interspaced Short Palindromic Repeats
ROC	Receiver operating characteristic
AUC	Area under the curve
