# Supplementary material for: Remodeling of the circRNA Landscape in Myocardial Infarction Integrates Nuclear Regulation, DNA Damage Response, and Cardiomyocyte Structural Pathways
Source: Biomolecules. 2026 Apr 14;16(4):578. doi: 10.3390/biom16040578 (PMC13113775; doi:10.3390/biom16040578)
Supplement: Supplementary file 1 [file biomolecules-16-00578-s001.zip › biomolecules-4230541-supplementary.pdf]

Supplementary Materials

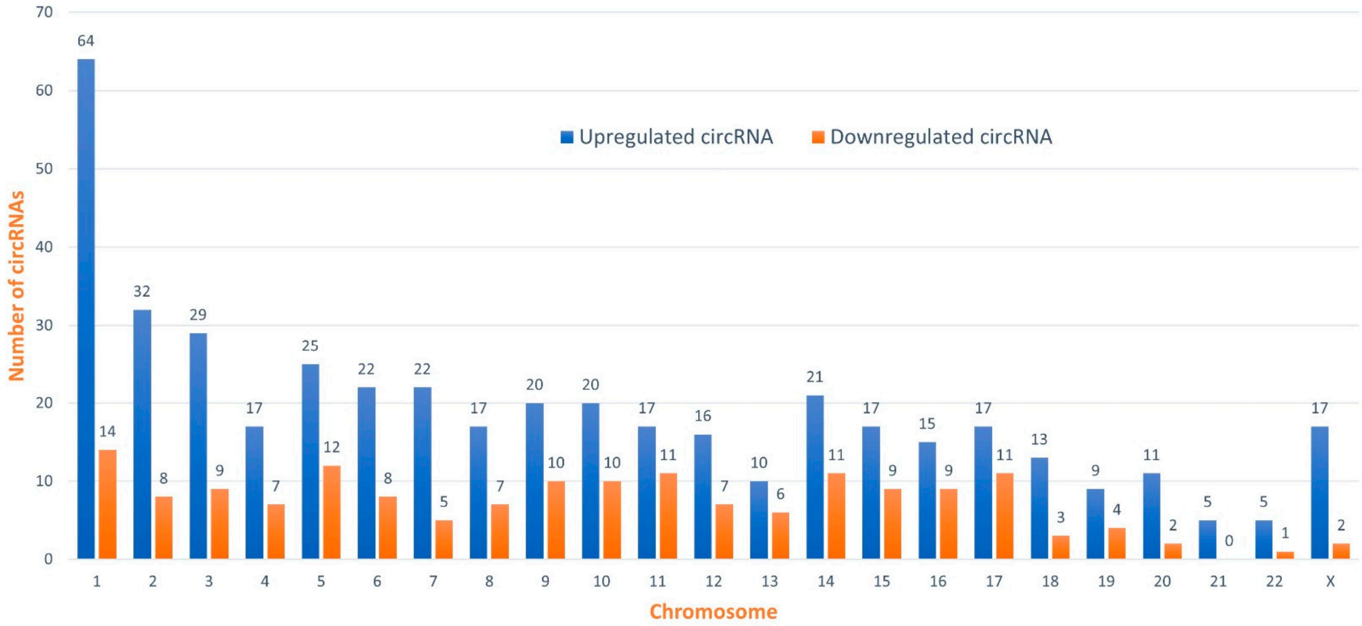

Supplementary Figure S1. Chromosomal distribution of differentially expressed circular RNAs (circRNAs). The figure shows the number of significantly upregulated and downregulated circRNAs mapped to host genes across individual chromosomes. Bars represent counts of circRNAs (not genes), categorized by direction of differential expression and chromosomal location. Differential expression significance thresholds were applied as described in the Methods

Supplementary Table S1. Case control demographic and clinical characteristics

	**Case**	**Control**
**Sample size, n**	**140**	**109**
**Gender (Male/Female)**	**116/24**	**71/38**
**Age, Mean ± SD**	**55.1± 11.25**	**20.17± 1.07**
**Age range (Years)**	**21-88**	**18-24**
**Unstable angina, n**	**3**	**0**
**Smokers, n**	**85**	**9**
**T2DM, n**	**85**	**0**
**HTN, n**	**87**	**0**
**Glucose Range (mg/dL)**	**77 - 432**	**NA**
**CVD Family History (%)**	**28.9**	**29.7**

**Troponin I Range (µg/L)**	**0.08 - 285.5**	**NA**
**HbA1c Range (%)**	**4.9 – 14.0**	**NA**

Supplementary Table S2. Top 50 upregulated circular RNA and their associated genes

S.No	circAtlas_ID	Gene name	Gene ID	Chromosome	circRNA_type	log2FoldChange	padj	AUC
1	hsa-CHD6_0001	CHD6	ENSG00000124177	20	exon	5.99	0.000	0.512
2	hsa-RPRD1B_0001	RPRD1B	ENSG00000101413	20	exon	5.68	0.000	0.624
3	hsa-LRBA_0191	LRBA	ENSG00000198589	4	exon	9.99	0.000	0.693
4	hsa-PASK_0004	PASK	ENSG00000115687	2	exon	6.54	0.000	0.745
5	hsa-TRAPPC9_0002	TRAPPC9	ENSG00000167632	8	exon	5.34	0.000	0.669
6	hsa-STRN3_0061	STRN3	ENSG00000196792	14	exon	7.10	0.000	0.68
7	hsa-STXBP3_0002	STXBP3	ENSG00000116266	1	exon	6.81	0.000	0.751
8	hsa-PAK1_0001	PAK1	ENSG00000149269	11	exon	8.61	0.000	0.676
9	hsa-SCAPER_0128	SCAPER	ENSG00000140386	15	intron	7.55	0.000	0.687
10	hsa-UBXN4_0004	UBXN4	ENSG00000144224	2	exon	5.76	0.000	0.537
11	hsa-DAAM1_0001	DAAM1	ENSG00000100592	14	exon	3.59	0.000	0.537
12	hsa-RCAN3_0002	RCAN3	ENSG00000117602	1	exon	5.68	0.000	0.715
13	hsa-AGGF1_0002	AGGF1	ENSG00000164252	5	exon	5.80	0.000	0.578
14	hsa-RANBP9_0044	RANBP9	ENSG00000010017	6	exon	4.42	0.000	0.812
15	hsa-RPS6KC1_0007	RPS6KC1	ENSG00000136643	1	exon	6.33	0.000	0.731
16	hsa-DIAPH1_0017	DIAPH1	ENSG00000131504	5	exon	5.53	0.000	0.741
17	hsa-FAM188A_0034	MINDY3	ENSG00000148481	10	exon	5.47	0.000	0.582
18	hsa-EIF4ENIF1_0030	EIF4ENIF1	ENSG00000184708	22	exon	4.51	0.000	0.669
19	hsa-DCAF5_0006	DCAF5	ENSG00000139990	14	exon	4.57	0.000	0.583
20	hsa-TRIM37_0130	TRIM37	ENSG00000108395	17	exon	5.87	0.000	0.631
21	hsa-CLHC1_0001	CLHC1	ENSG00000162994	2	exon	9.03	0.000	0.698
22	hsa-LCORL_0030	LCORL	ENSG00000178177	4	exon	7.22	0.000	0.714
23	hsa-RBM23_0002	RBM23	ENSG00000100461	14	exon	2.60	0.000	0.679

24	hsa-SLC45A4_0001	SLC45A4	ENSG00000022567	8	exon	3.72	0.000	0.524
25	hsa-SP140L_0001	SP140L	ENSG00000185404	2	exon	4.85	0.000	0.727
26	hsa-UBXN7_0009	UBXN7	ENSG00000163960	3	exon	3.48	0.000	0.621
27	hsa-MFAP1_0001	MFAP1	ENSG00000140259	15	exon	5.18	0.000	0.508
28	hsa-MCU_0001	MCU	ENSG00000156026	10	exon	2.30	0.000	0.69
29	hsa-TIMMDC1_0002	TIMMDC1	ENSG00000113845	3	exon	6.36	0.001	0.711
30	hsa-UXS1_0028	UXS1	ENSG00000115652	2	exon	2.53	0.001	0.743
31	hsa-CNN2_0003	CNN2	ENSG00000064666	19	exon	3.24	0.001	0.524
32	hsa-SEPT11_0028	SEPTIN11	ENSG00000138758	4	exon	7.15	0.001	0.726
33	hsa-PKHD1L1_0001	PKHD1L1	ENSG00000205038	8	exon	4.27	0.001	0.709
34	hsa-TPM4_0006	TPM4	ENSG00000167460	19	exon	4.22	0.001	0.628
35	hsa-CDC73_0017	CDC73	ENSG00000134371	1	exon	4.86	0.001	0.74
36	hsa-PRKCB_0006	PRKCB	ENSG00000166501	16	exon	7.66	0.001	0.61
37	hsa-IKBKB_0001	IKBKB	ENSG00000104365	8	exon	3.22	0.001	0.654
38	hsa-ADAM17_0001	ADAM17	ENSG00000151694	2	exon	5.48	0.001	0.677
39	hsa-PGS1_0016	PGS1	ENSG00000087157	17	intron	6.03	0.001	0.601
40	hsa-ZNRD1-AS1_0002	POLR1HASP	ENSG00000204623	6	exon	6.11	0.001	0.595
41	hsa-CD99L2_0001	CD99L2	ENSG00000102181	X	exon	4.90	0.001	0.712
42	hsa-LARP1B_0006	LARP1B	ENSG00000138709	4	exon	7.04	0.001	0.75
43	hsa-MDM2_0003	MDM2	ENSG00000135679	12	exon	4.94	0.001	0.728
44	hsa-SRSF4_0027	SRSF4	ENSG00000116350	1	exon	3.02	0.001	0.539
45	hsa-ME2_0002	ME2	ENSG00000082212	18	exon	5.54	0.001	0.759
46	hsa-DNM3_0037	DNM3	ENSG00000197959	1	intron	4.92	0.001	0.716
47	hsa-TRNAU1AP_0001	TRNAU1AP	ENSG00000180098	1	exon	5.20	0.001	0.797
48	hsa-ZC3H6_0027	ZC3H6	ENSG00000188177	2	exon	2.79	0.001	0.687
49	hsa-GSE1_0005	GSE1	ENSG00000131149	16	exon	2.01	0.001	0.517
50	hsa-ZC3H14_0046	ZC3H14	ENSG00000100722	14	exon	5.80	0.001	0.648

Supplementary table S3: Top 50 downregulated circular RNA and their associated genes

S.No	circAtlas_ID	Gene name	Gene ID	Chromosome	circRNA_type	log2FoldChange	padj	AUC
1	hsa-SEPT11_0030	SEPTIN11	ENSG00000138758	4	exon	-7.97	0.000	0.693
2	hsa-CENPK_0003	CENPK	ENSG00000123219	5	exon	-7.87	0.000	0.748
3	hsa-FAM208A_0056	TASOR	ENSG00000163946	3	exon	-5.72	0.000	0.76
4	hsa-COPS2_0003	COPS2	ENSG00000166200	15	exon	-7.27	0.000	0.744
5	hsa-HIF1A_0002	HIF1A	ENSG00000100644	14	exon	-5.91	0.000	0.812
6	hsa-SUZ12_0049	SUZ12	ENSG00000178691	17	exon	-6.73	0.000	0.803
7	hsa-CDKN3_0007	CDKN3	ENSG00000100526	14	exon	-5.72	0.000	0.785
8	hsa-TXNL4B_0001	TXNL4B	ENSG00000140830	16	exon	-3.70	0.001	0.741
9	hsa-PAPOLA_0001	PAPOLA	ENSG00000090060	14	exon	-4.01	0.001	0.658
10	hsa-PNRC1_0001	PNRC1	ENSG00000146278	6	exon	-4.57	0.001	0.857
11	hsa-ST8SIA4_0002	ST8SIA4	ENSG00000113532	5	exon	-6.55	0.001	0.715
12	hsa-BRCA1_0011	BRCA1	ENSG00000012048	17	exon	-6.49	0.001	0.72
13	hsa-WBSCR22_0002	BUD23	ENSG00000071462	7	exon	-5.80	0.001	0.723
14	hsa-GNAQ_0013	GNAQ	ENSG00000156052	9	exon	-3.45	0.001	0.77
15	hsa-WDR37_0007	WDR37	ENSG00000047056	10	intron	-5.84	0.002	0.797
16	hsa-RSL1D1_0001	RSL1D1	ENSG00000171490	16	exon	-4.91	0.002	0.776
17	hsa-TLK1_0013	TLK1	ENSG00000198586	2	exon	-5.07	0.002	0.794
18	hsa-CDK19_0044	CDK19	ENSG00000155111	6	intron	-7.06	0.002	0.726
19	hsa-RAB2A_0002	RAB2A	ENSG00000104388	8	exon	-5.08	0.002	0.831
20	hsa-DHDDS_0001	DHDDS	ENSG00000117682	1	exon	-4.82	0.002	0.747
21	hsa-ZNF426_0001	ZNF426	ENSG00000130818	19	exon	-5.91	0.002	0.743
22	hsa-MTDH_0009	MTDH	ENSG00000147649	8	exon	-7.09	0.002	0.7
23	hsa-DNM1L_0012	DNM1L	ENSG00000087470	12	exon	-6.29	0.002	0.757
24	hsa-ARHGAP32_0002	ARHGAP32	ENSG00000134909	11	exon	-4.40	0.003	0.734
25	hsa-MRE11A_0003	MRE11	ENSG00000020922	11	exon	-6.17	0.003	0.725
26	hsa-APBB2_0002	APBB2	ENSG00000163697	4	exon	-6.43	0.003	0.705

27	hsa-ATP6V1H_0003	ATP6V1H	ENSG00000047249	8	exon	-4.66	0.003	0.758
28	hsa-DENND4C_0003	DENND4C	ENSG00000137145	9	exon	-5.88	0.003	0.748
29	hsa-LSM14A_0001	LSM14A	ENSG00000257103	19	exon	-5.68	0.004	0.758
30	hsa-CNOT2_0055	CNOT2	ENSG00000111596	12	exon	-5.02	0.004	0.714
31	hsa-SH3GLB1_0003	SH3GLB1	ENSG00000097033	1	exon	-5.05	0.004	0.735
32	hsa-WDR44_0002	WDR44	ENSG00000131725	X	exon	-4.35	0.004	0.76
33	hsa-ZNF85_0001	ZNF85	ENSG00000105750	19	exon	-6.16	0.004	0.794
34	hsa-CSPP1_0027	CSPP1	ENSG00000104218	8	exon	-4.77	0.004	0.702
35	hsa-PICALM_0086	PICALM	ENSG00000073921	11	exon	-5.47	0.005	0.725
36	hsa-UHRF2_0050	UHRF2	ENSG00000147854	9	intron	-5.45	0.005	0.778
37	hsa-CSGALNACT2_0001	CSGALNACT2	ENSG00000169826	10	exon	-5.90	0.005	0.701
38	hsa-MTRF1_0001	MTRF1	ENSG00000120662	13	exon	-5.23	0.005	0.802
39	hsa-TTC1_0002	TTC1	ENSG00000113312	5	exon	-5.42	0.005	0.725
40	hsa-TOP3A_0006	TOP3A	ENSG00000177302	17	exon	-5.50	0.006	0.786
41	hsa-RAB1A_0009	RAB1A	ENSG00000138069	2	exon	-6.28	0.006	0.752
42	hsa-PALMD_0003	PALMD	ENSG00000099260	1	exon	-5.99	0.006	0.657
43	hsa-SIPA1L1_0011	SIPA1L1	ENSG00000197555	14	exon	-3.01	0.007	0.714
44	hsa-BRF1_0001	BRF1	ENSG00000185024	14	exon	-4.62	0.007	0.677
45	hsa-USP49_0001	USP49	ENSG00000164663	6	exon	-4.16	0.007	0.74
46	hsa-SCAF8_0002	SCAF8	ENSG00000213079	6	exon	-4.12	0.007	0.795
47	hsa-POLB_0016	POLB	ENSG00000070501	8	exon	-5.60	0.007	0.697
48	hsa-STK38_0019	STK38	ENSG00000112079	6	exon	-5.46	0.007	0.645
49	hsa-GDI2_0001	GDI2	ENSG00000057608	10	exon	-2.84	0.008	0.756
50	hsa-SKA3_0006	SKA3	ENSG00000165480	13	exon	-4.63	0.008	0.79
